# The Role of Feeding Characteristics in Shaping Gut Microbiota Composition and Function of Ensifera (Orthoptera)

**DOI:** 10.3390/insects13080719

**Published:** 2022-08-10

**Authors:** Xiang Zheng, Qidi Zhu, Meng Qin, Zhijun Zhou, Chunmao Liu, Liyuan Wang, Fuming Shi

**Affiliations:** 1 Key Laboratory of Zoological Systematics and Application of Hebei Province, College of Life Sciences, Hebei University, Baoding 071002, China; 2 Laboratory of Enzyme Preparation, Hebei Research Institute of Microbiology Co., Ltd., Baoding 071051, China; 3 College of Life Sciences, Hebei Agricultural University, Baoding 071001, China

**Keywords:** metagenomic, gut microbiota, feeding habits, KEGG, CAZymes, Ensifera

## Abstract

**Simple Summary:**

Feeding habits were the main factor affecting the gut microbial community structure of Ensifera (Insecta: Orthoptera). The gut microbial communities of Ensifera with different feeding habits were significantly different, as insects with more diverse feeding habits had gut microorganisms with less specific functions. However, feeding habits are not the only factors that affect the gut microbial community structure of Ensifera. Factors related to energy and nutrition acquisition also affect them, such as the abundance of some microbial functional genes unrelated to feeding habits but related to survival.

**Abstract:**

Feeding habits were the primary factor affecting the gut bacterial communities in Ensifera. However, the interaction mechanism between the gut microbiota and feeding characteristics is not precisely understood. Here, the gut microbiota of Ensifera with diverse feeding habits was analyzed by shotgun metagenomic sequencing to further clarify the composition and function of the gut microbiota and its relationship with feeding characteristics. Our results indicate that under the influence of feeding habits, the gut microbial communities of Ensifera showed specific characteristics. Firstly, the gut microbial communities of the Ensifera with different feeding habits differed significantly, among which the gut microbial diversity of the herbivorous *Mecopoda niponensis* was the highest. Secondly, the functional genes related to feeding habits were in high abundance. Thirdly, the specific function of the gut microbial species in the omnivorous *Gryllotalpa orientalis* showed that the more diverse the feeding behavior of Ensifera, the worse the functional specificity related to the feeding characteristics of its gut microbiota. However, feeding habits were not the only factors affecting the gut microbiota of Ensifera. Some microorganisms’ genes, whose functions were unrelated to feeding characteristics but were relevant to energy acquisition and nutrient absorption, were detected in high abundance. Our results were the first to report on the composition and function of the gut microbiota of Ensifera based on shotgun metagenomic sequencing and to explore the potential mechanism of the gut microbiota’s association with diverse feeding habits.

## 1. Introduction

Insects are the main group of arthropods, as well as one of the most diverse groups of animals on earth [1,2]. The diversification and successful evolution of insects were closely related to the symbiotic relationship between them and gut microorganisms in the long-term coevolution process [3,4]. In particular, many symbiotic microorganisms have explicitly adapted to insects as hosts and may participate in numerous metabolic activities. Microorganisms play crucial roles in acquiring and absorbing nutrients [5], secreting digestive enzymes [6], secreting immune-related compounds [7], enhancing pathogen resistance [8,9], and influencing social interactions [10], among other roles. However, identifying these microorganisms and determining their function remains challenging [11].

Microbiota is studied using both culture-dependent [12] and culture-independent [13] methods. However, due to the limitations associated with culture-dependent techniques, most gut microorganisms remain uncultured, limiting the possibility of describing the gut microbial community characteristics through culture techniques [14]. With the development of high-throughput sequencing (HTS) technology, significant progress was made in studying the gut microbiota through molecular biotechnology [15,16,17]. Furthermore, it allowed us to better understand the microbiota’s structure, function, and diversity without culturing [18]. The 16S rRNA gene and shotgun metagenomic sequencing methods, with the characteristic of being microbial culture-independent, are the two main HTS tools that provide insights into microbial community composition and function [18,19]. However, 16S rRNA gene sequencing has limited genomic scope and amplification biases toward particular taxonomic groups. Therefore, no data are provided regarding the functional capacity of a microbial community [20]. More recently, shotgun metagenomic sequencing was applied to describe the viruses, bacteria, archaea, and eukaryotes that compose a given microbiota and explore their implications in metabolism [21,22,23].

Many factors can influence insect gut microbial community structure, such as feeding behavior, host taxonomy, life stage, and host environment [2,24,25,26,27]. A previous study found that the feeding characteristics affect the structure of the insect gut microbial community, which in turn affects the growth and development of the insect [28]. Studies also found that changes in the ecological environment will affect the type of food consumed, leading to changes in the gut microbial community of insects, which may harm their survival [29]. Meanwhile, gut microorganisms play an essential role in promoting the digestion and absorption of the host. Some insects evolved to use lignocellulose substrates as energy by using microbial metabolites [30]. Some symbiotic microorganisms secrete gut enzymes through the hydrolysis of ingested plant cell wall polysaccharides [31]. In other words, the host’s feeding behavior and gut microbial community interact. However, the mechanism by which the host feeding behavior modifies the gut microbial community is unclear.

The suborder Ensifera (Insecta: Orthoptera) records about 7971 species and 19 subfamilies, many of which are endemic to China. Chinese Ensifera includes species of katydids, crickets, mole crickets, and wetas, with high species diversity and diversified feeding habits [32,33,34]. These species are major agricultural and forestry pests and potential resources for biological control. Ensifera is thoroughly studied taxonomically and phylogenetically, providing resources for studying the gut microbiota [35]. In our previous study, the 16S rRNA amplicon sequencing technology was used for sequence analysis of the gut bacterial communities of 12 species of Ensifera from 5 families. It was found that feeding habits were the primary factors affecting the gut bacterial communities, and samples from different taxa with the same feeding habit showed similar gut bacterial community structures [36]. However, the microbial composition and functional diversity of the gut microbiota in Ensifera, especially the potential mechanism of the relationship between the function of the gut microbiota and feeding habits, remains undetermined. Here, we performed shotgun metagenomic sequencing of three selected Ensifera species from our previous study mentioned above. Then, we directed our attention to the composition of the gut microbial community to better describe the relationship between specific microbiota functions and feeding characteristics.

## 2. Materials and Methods

### 2.1. Sample Collection and DNA Extraction

The adult Ensifera species were collected from two national nature reserve sites and one farmland site in China in 2019 (Table 1), and were immediately submerged in 99% (v/v) ethanol after capture until identification and dissection [37]. Based on morphological characteristics, these samples were identified by the Katydids Laboratory of Hebei University as *Mecopoda niponensis* (Mec) belonging to the family Tettigoniidae, *Ocellarnaca emeiensis* (Oce) belonging to the family Gryllacrididae, and *Gryllotalpa orientalis* (Gry) belonging to the family Gryllotalpidae, which were characterized as herbivore, carnivore, and omnivore feeding habits, respectively. The Department of Forestry of Guangxi Zhuang Autonomous Region approved our entry to the Daming Mountains National Nature Reserve to collect insect samples. The Zhejiang Tianmu Mountains National Nature Reserve Administration approved our entry to the Tianmu Mountains National Nature Reserve to collect insect samples. Samples from Hebei Province were collected from farmland. No endangered or protected species were used in this study.

Gut dissection was performed as follows: Each insect species (*n* = 15) was gently dissected by collecting the midgut and hindgut with gut contents under sterilized conditions [38,39,40], and 5 guts were randomly pooled together as one biological replicate sample. Then each sample was processed to extract DNA individually, using the TIANamp Stool DNA Kit (TIANGEN, Beijing, China) according to the manufacturer’s protocols [13,41]. Sample blanks consisted of unused swabs processed through DNA extraction and were tested to contain no DNA amplicons. Following the extraction, the total DNA in each gut sample was measured using a NanoDrop 2000 spectrophotometer (Thermo Fisher Scientific, Waltham, MA, USA) and stored at −20 °C until sequencing by LC-Bio Technologies Co., Ltd., Hangzhou, China. 

### 2.2. DNA Library Construction

After the DNA library was constructed and passed the quality test, Novaseq 6000 was used for high-throughput sequencing. The sequencing mode was PE150. The sequencing kit was the TruSeq Nano DNA LT Library Preparation Kit (FC-121-4001, Illumina, San Diego, CA, USA) and was fragmented by dsDNA Fragmentase (NEB, M0348S, Ipswich, MA, USA) at 37 °C for 30 min. The construction began with fragmented cDNA generated using a combination of fill-in reactions and exonuclease activity, and size selection was performed with the provided sample purification beads. The A-base was added to each strand’s blunt ends to prepare them for ligation to the indexed adapters. Each adapter contained a T-base overhang for ligating the adapter to the A-tailed fragmented DNA and the full complement of the sequencing primer hybridization sites for single, paired-end, and indexed reads. Single- or dual-index adapters were ligated to the fragments [42]. Then, they were amplified with PCR using the following conditions: initial denaturation at 95 °C for 3 min; 8 cycles of denaturation at 98 °C for 15 s, annealing at 60 °C for 15 s, and extension at 72 °C for 30 s; and then final extension at 72 °C for 5 min.

### 2.3. Metagenomics Data Assembly and Analysis

Raw sequencing data were removed from the connector sequences to obtain valid reads. Firstly, sequencing adapters were removed using cutadapt (v 1.9, https://github.com/marcelm/cutadapt, accessed on 8 November 2020). Secondly, low-quality reads were trimmed by fqtrim (v 0.94, http://ccb.jhu.edu/software/fqtrim/, accessed on 8 November 2020) using a sliding window algorithm. Thirdly, reads were aligned to the host genome using bowtie2 (v2.2.0, http://bowtie-bio.sourceforge.net/bowtie2/, accessed on 10 November 2020) to remove host contamination. After data preprocessing, the quality-filtered reads were de novo assembled into contigs using IDBA-UD (v1.1.1, http://i.cs.hku.hk/~alse/hkubrg/projects/idba_ud/, accessed on 12 November 2020) [43]. QUAST (v3.2, St. Petersburg Academic University of the Russian Academy of Sciences, St Petersburg, Russian) was used to visualize the mapping of the genome bin contigs against the closest reference genome [44]. MetaGeneMark (v3.26, Georgia Tech, Atlanta, GA, USA) was used to predict the coding region (CDS) of assembled contigs (≥500 bp), and the CDS sequences less than 100 NT were filtered. Then, CD-HIT (v4.6.1, http://www.bioinformatics.org/cd-hit/, accessed on 12 November 2020) was used to remove redundancy, and 95% of identity and 90% of coverage were used to cluster [45]. Then, bowtie2 (v2.2.0, http://bowtie-bio.sourceforge.net/bowtie2/, accessed on 13 November 2020) was used to compare each sample’s clean data to the gene sequence, and the number of reads was calculated. The genome sequence with fewer reads (≤2) was filtered out to obtain unigenes for subsequent analysis. The taxonomy was annotated by searching against the NR_ Meta database (blastp, evalue ≤ 1 × 10^−5^) using DIAMOND (v0.9.14, Max Planck Institute for Biology, Tübingen, Germany) [46]. Combined with NCBI’s species classification system, species annotation information at different taxonomic levels was obtained [47]. Similarly, the unigenes’ functional annotation by the KEGG and CAZymes databases was obtained. 

The reference genomes from *Teleogryllus occipitalis* (https://www.ncbi.nlm.nih.gov/genome/?term=GCA_011170035.1, accessed on 10 November 2020) and *Laupala kohalensis* (https://www.ncbi.nlm.nih.gov/genome/?term=GCA_002313205.1, accessed on 10 November 2020), which are closely related to the samples in this study, were retrieved from the NCBI database.

### 2.4. Statistical Analyses

Statistical analyses were carried out via R software (v4.1.2, http://cran.r-project.org, accessed on 25 May 2022) [48]. The alpha diversity was calculated using species-level annotation information statistics, and differences between the groups were assessed using the Kruskal–Wallis test [47], with *p* < 0.05 as a significant difference. The beta diversity of PCoA was tested with analysis of similarities (ANOSIM) [49] to analyze the differences between samples, with *p* < 0.05 as a significant difference. LEfSe analysis [50] (LDA score > 4) was used to find the biomarkers of the samples, with *p* < 0.05 as a significant difference. UpSet analysis (threshold > 0.1) was used to show each sample’s shared and unique microorganism. Unigenes were compared with the KEGG [51] and CAZymes databases [52,53] (blastp, evalue ≤ 1 × 10^−5^) to obtain the annotation enrichment of each database. The statistical analysis was carried out by pairwise comparisons with Welch’s *t*-test [54] at KEGG levels 1 and 2, and CAZy level 2, with *p* < 0.05 as a significant difference. In addition, among the KO entry metabolic pathways of different feeding habits, the pathways related to carbohydrate metabolism, lipid metabolism, and amino acid metabolism with significant differences (*p* < 0.05) were compared and analyzed. The genes with 100% identity in the CAZymes database were screened, and their annotated species information, as well as the annotated enzyme information of CAZy level 2, was analyzed for correlation. 

## 3. Results

In a previous study, we investigated the diversity of the gut bacterial communities of Ensifera from twelve species of five families. We found that feeding characteristics were the main factor affecting the structure of the gut bacterial communities. The gut bacterial communities’ structure in Ensifera, from different taxa but with the same feeding habit, was similar [36]. Therefore, we selected three Ensifera species (Mec, Oce, and Gry) with high bacterial community diversity and minor intraspecific differences from the above samples to explore the similarities and differences in the gut microbiota’s composition and function mediated by feeding characteristics. 

After extracting DNA from each gut sample, the collected samples were analyzed by shotgun metagenomic sequencing. Since the raw sequencing data may contain splice sequences and a certain proportion of low-quality data, clean data for subsequent analysis could be obtained after quality trimming and host genome filtering. The preprocessing results are shown in Table S1. Gry obtained the most sequencing reads, with a value of 85,662,373, followed by Oce and Mec, with 82,575,455 and 79,442,693, respectively. After data preprocessing, IDBA-UD was assembled using a single sample, and QUAST evaluated the assembly results. The assembly results are shown in Table S2. A co-assembly of samples of Mec, Oce, and Gry generated 71,476, 129,921, and 417,677 contigs, respectively.

### 3.1. The Diversity and Composition of the Gut Microbiota

**Figure 1:**
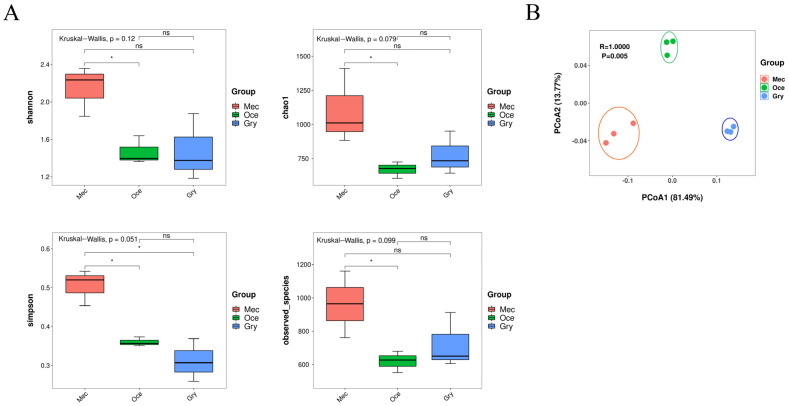
Gut microbiota composition of the three species. Relative abundance of gut microbial composition, (**A**) Alpha diversity of the gut microbial community based on Shannon, Chao1, Simpson, and observed_species (ns, *p* > 0.05; *, *p* < 0.05). (**B**) Beta diversity of PCoA analysis based on Bray–Curtis distances to compare differences between species.

The alpha diversity analysis of the three species showed that the richness and diversity of the gut microbiota in Mec were the highest. In contrast, Gry and Oce’s gut microbiota was with high diversity similarity. There was no significant difference in the alpha diversity index among the three species, however, with a significant difference between Mec and Oce (Kruskal–Wallis, *p* < 0.05) (Figure 1A). Based on Bray–Curtis distances, the beta diversity from the principal coordinate analysis (PCoA) showed significant differences in gut microbial structure among the three species (ANOSIM: R = 1, *p* = 0.005). Meanwhile, the sample of each feeding habit clustered together indicated that the intraspecific similarity of the species was high (Figure 1B).

The genes obtained from preprocessing were compared in the NR database (blastp, evalue ≤ 1 × 10^−5^), and then the species annotation at different taxonomic levels was obtained. Subsequently, the species abundance at each taxonomic level was obtained by combining the species classification with the gene abundance. The classified sequences were assigned to bacteria, eukaryotes, viruses, and archaea. Among the annotated classified species, the abundance of bacteria was the highest, and the bacterial abundances of Mec, Oce, and Gry were 81.91%, 88.71%, and 86.03%, respectively. Viruses were the next most abundant in the gut microbiota of Mec and Gry, while eukaryotes were the next most abundant in Oce (Table S3).

At the phylum level, the structure of the gut microbiota of the three species was different. For instance, Firmicutes were the highest abundance of microbiota in Gry, Whereas Proteobacteria had the highest abundance of microbiota in Mec and Oce. Although the bacterial abundance of Mec and Oce occupied the first place, there were several eukaryotic phyla with high abundances, such as Mucoromycota and Basidiomycota. At the species level, the dominant species of the three species were quite different. In particular, *Intestinimonas massiliensis*, with the highest abundance in Gry, were not detected in Mec and Oce. Meanwhile, both Gry and Oce had a high abundance of microbiota as dominant species (Gry: *Intestinimonas massiliensis*; Oce: *Lactococcus lactis*). However, although Mec had a highly diverse microbiota, no dominant taxonomic group was detected (Table 2).

**Figure 2:**
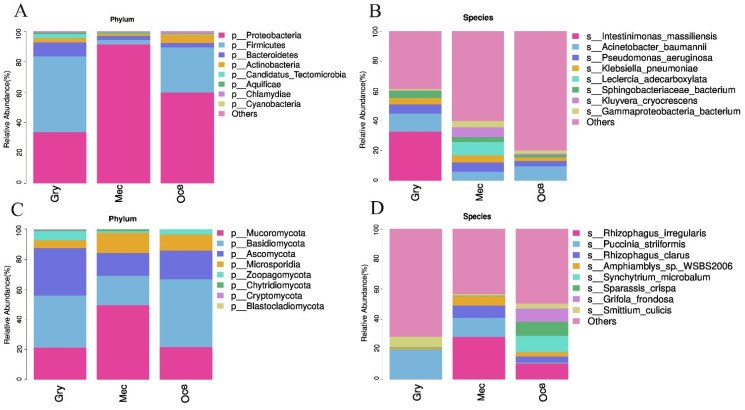
A stacked bar chart revealing the relative abundance of gut bacterial and eukaryotic composition at the (**A**,**C**) phylum and (**B**,**D**) species levels. The results show the phylum and species of the gut microbiota with the highest abundance.

At the phylum level, the main microbial phyla of bacteria (Figure 2A), eukaryotes (Figure 2C), and viruses (Figure S1A) were the same, except archaea (Figure S1C). However, the relative abundance and the dominant microbial components differed. Furthermore, the microbial composition of viruses and archaea was low diversity, and few viruses were detected in Oce. At the species level, bacteria (Figure 2B) specific to three species were found in the dominant bacterial populations, such as *Intestinimonas massiliensis* in Gry, *Leclercia adecarboxylata* in Mec, and *Lactococcus lactis* in Oce. However, although more specific eukaryotes with high abundance (Figure 2D) were found in Gry (*Metarhizium majus* and *Endogone* sp. *FLAS-F59071*) and Oce (*Synchytrium microbalum* and *Sparassis crispa*), eukaryotes with high abundance (*Rhizophagus irregularis*, *Puccinia striiformis* and *Rhizophagus clarus*) in Mec were also detected in other species. The composition and structure of gut viruses (Figure S1B) were completely different. Archaea (Figure S1D) accounted for less than 0.2% of Ensifera’s gut microbiota and showed little difference except that Mec was the only one containing *Thaumarchaeota archaeon*. 

### 3.2. The Characteristics of the Gut Microbiota 

**Figure 3:**
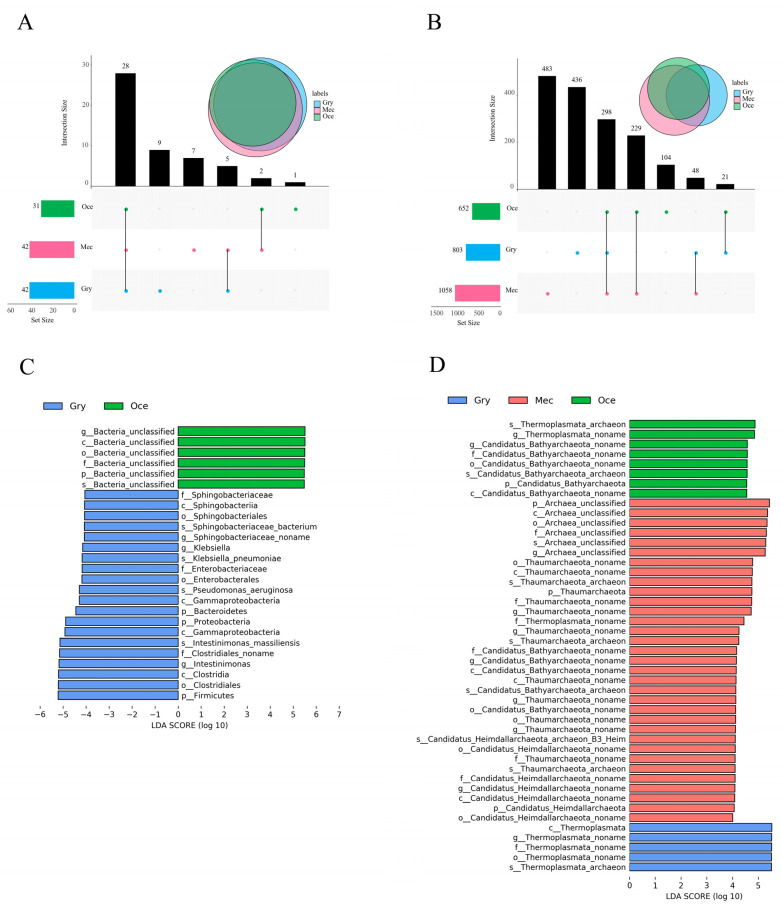
Characteristics of gut microbiota composition. UpSet analysis of the shared and unique microorganisms at the (**A**) phylum and (**B**) species levels between species. Distribution diagram of the LEfSe analysis based on the LDA score of (**C**) bacteria and (**D**) archaea to screen the biomarkers.

Based on the analysis of the three species’ gut microbiota structure and diversity, the shared and unique microorganisms at the phylum and species level were displayed by UpSet analysis (threshold > 0.1). At the phylum level (Figure 3A), the number of shared microorganisms (28) of the three species was more than unique microorganisms, and most of them were in high abundance. However, the number of unique microorganisms of Gry was nine, while for Mec and Oce, it was seven and one, respectively. At the species level (Figure 3B), the numbers of unique microorganisms of Mec (483) and Gry (436) were more than the number of shared microorganisms (298), which overlapped in the three species. The number of unique microorganisms in Oce was about 1/4 of that in the other species. From the relationship between the two species, the number of microorganisms that overlapped among Mec and Oce was 527, which was more than the number of unique microorganisms. 

To further explore the differences in the gut microbiota, LEfSe analysis (LDA score > 4, *p* < 0.05) was used to find the biomarkers in bacteria (Figure 3C), eukaryotes (Figure S2A), viruses (Figure S2B), and archaea (Figure 3D), with significant abundance differences among the three species. In terms of the biomarker numbers, there was little difference in gut eukaryotes and viruses among the samples. However, with significant differences between gut bacteria and archaea. Among them, no biomarker was found in the gut bacteria of Mec, whereas the number of biomarkers in the gut archaea of Mec was the highest. In terms of the biomarker species, bacteria and viruses were the main biomarkers of Gry, mainly including Firmicutes, Proteobacteria, Bacteroidetes, and *Cotesia sesamiae bracovirus*. Archaea were the main biomarkers of Mec, mainly Thermoplasmata. 

### 3.3. Metabolic Potential Functions of Gut Microbiota According to the KEGG Database

**Figure 4:**
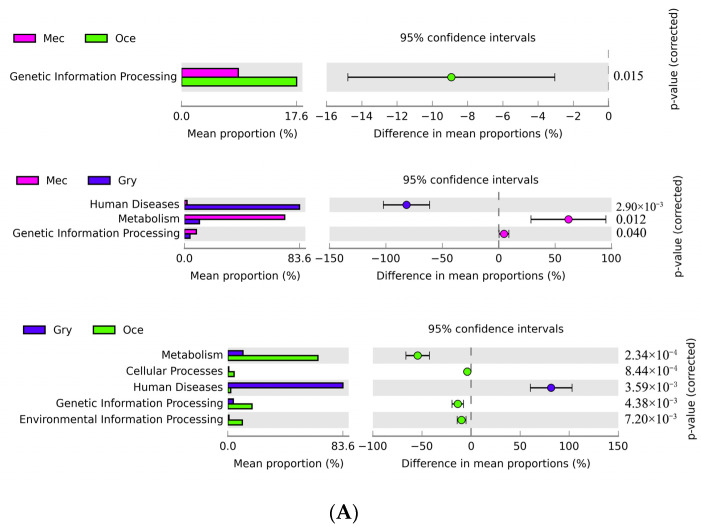

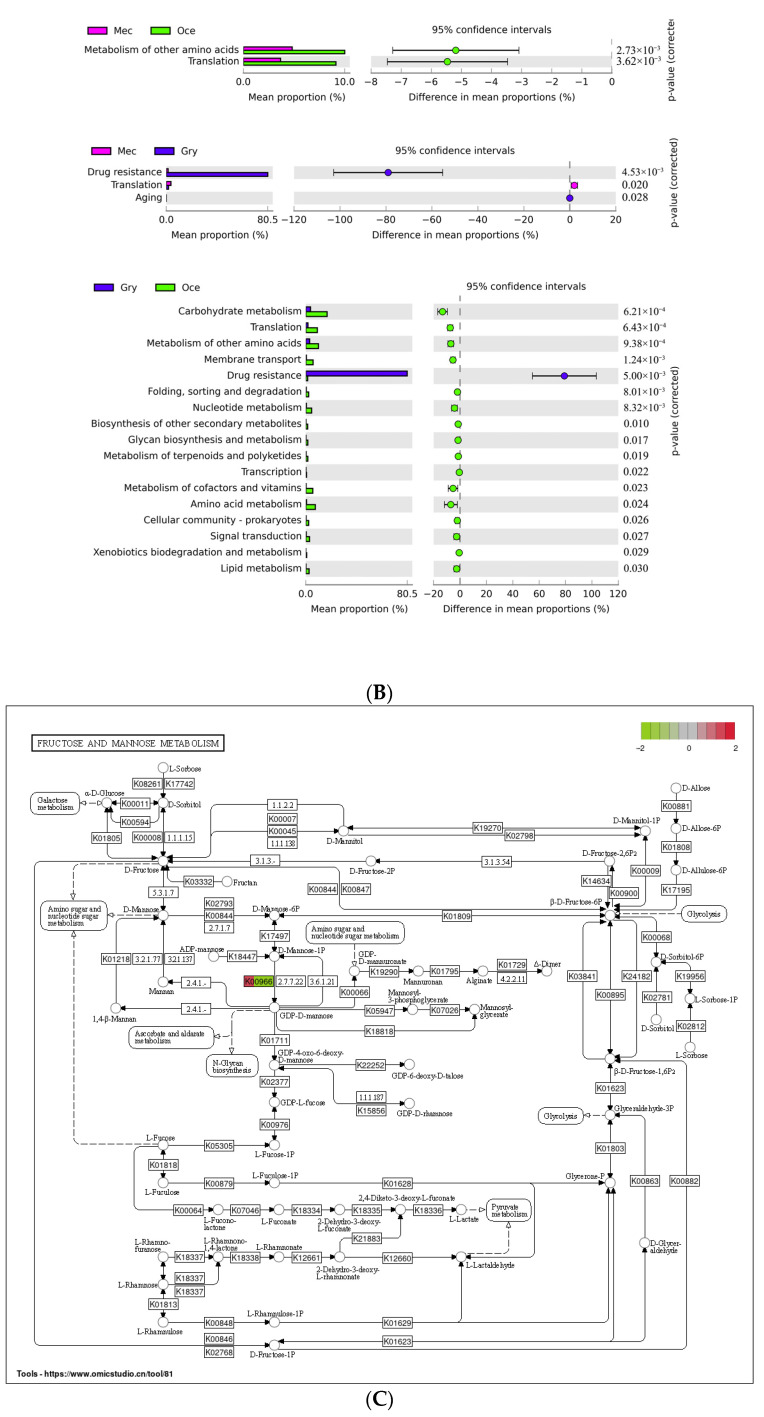

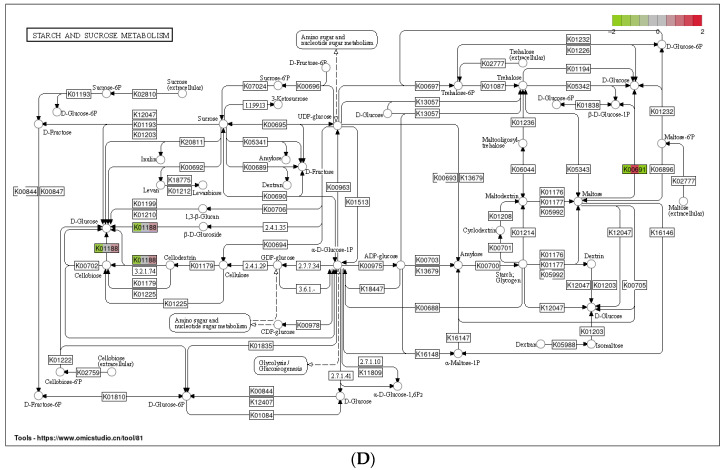
Functional and pathway annotation comparison of the genes assigned to the KEGG database. (**A**) Function annotation at KEGG level 1. (**B**) Function annotation at KEGG level 2. (**C**) Gene abundance comparison in the fructose and mannose metabolism pathway. (**D**) Gene abundance comparison in the starch and sucrose metabolism pathway. Notes for C and D: The genes belonging to the three samples with significant differences in the metabolic pathway map are marked by a color, in which Mec, Oce, and Gry are from left to right. The red to green color represents the high to low gene abundance. (https://www.omicstudio.cn/tool, accessed on 22 May 2022).

The functional annotation of gut microbiota in the KEGG database was investigated, and these annotated biological functions were divided into six categories. In Mec and Oce, more than 65% of the genes were mapped onto metabolism, followed by genes mapped onto genetic information processing and environmental information processing. However, nearly 85% of the genes were mapped onto human diseases in Gry, followed by genes related to metabolism (Table 3). At KEGG level 1, genetic information processing was the dominant pathway in Oce through pairwise comparison (Welch’s *t*-test, *p* < 0.05), and human diseases were the dominant pathway in Gry (Figure 4A). At KEGG level 2, metabolism of other amino acids and translation was the dominant pathway in Oce through pairwise comparison (Welch’s *t*-test, *p* < 0.05), and drug resistance was the dominant pathway in Gry (Figure 4B). No metabolic pathway significantly higher than the other species was found in Mec.

In order to deeply explore the relationship between the metabolic function of the gut microbiota and feeding characteristics, we performed a difference analysis on KEGG ORTHOLOGY (KO) database entry. Then, the carbohydrate metabolism, lipid metabolism, and amino acid metabolism pathways, which were significantly different (*p* < 0.05) and related to food digestion and absorption, were further explored. Among them, the gene abundance of Mec in the carbohydrate metabolism pathway was higher than that of the other species, such as fructose and mannose metabolism (ID: map00051) (Figure 4C) and galactose metabolism (ID: map00052) (Figure S3A). However, in starch and sucrose metabolism (ID: map00500) (Figure 4D), Gry showed the most substantial ability to convert cellulose into glucose, and Oce showed the most substantial ability to convert maltose into glucose. Interestingly, there was no high abundance of sequences related to lipid metabolism on Oce. In amino acid metabolism, it was found that Mec and Oce have high gene abundance in the biosynthesis of some amino acids, such as arginine biosynthesis (ID: map00220) (Figure S3B) in Oce and phenylalanine, tyrosine and tryptophan biosynthesis (ID: map00400) (Figure S3C) in Mec. Importantly, we did not find a high abundance of genes related to nutrition in Gry. However, we found a high gene abundance (k02172: bla regulator protein blaR1) involved in beta lactam resistance (ID: map01501) (Figure S3D) in Gry.

### 3.4. Metabolic Potential Functions of Gut Microbiota According to CAZymes Database

**Figure 5:**
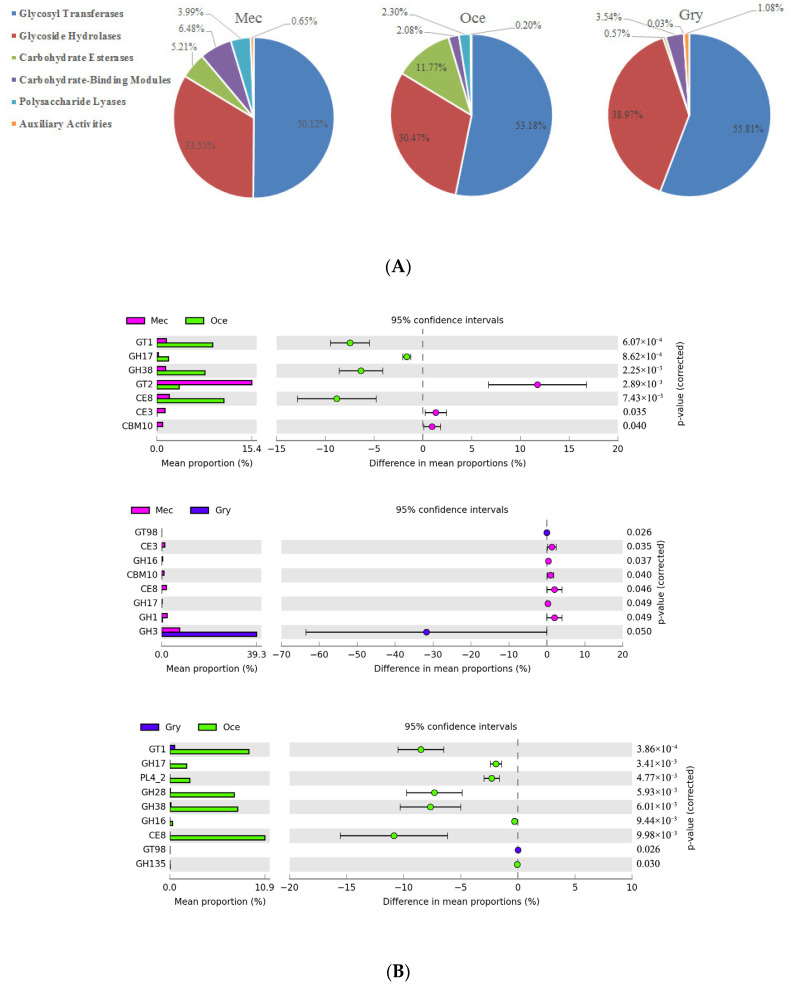

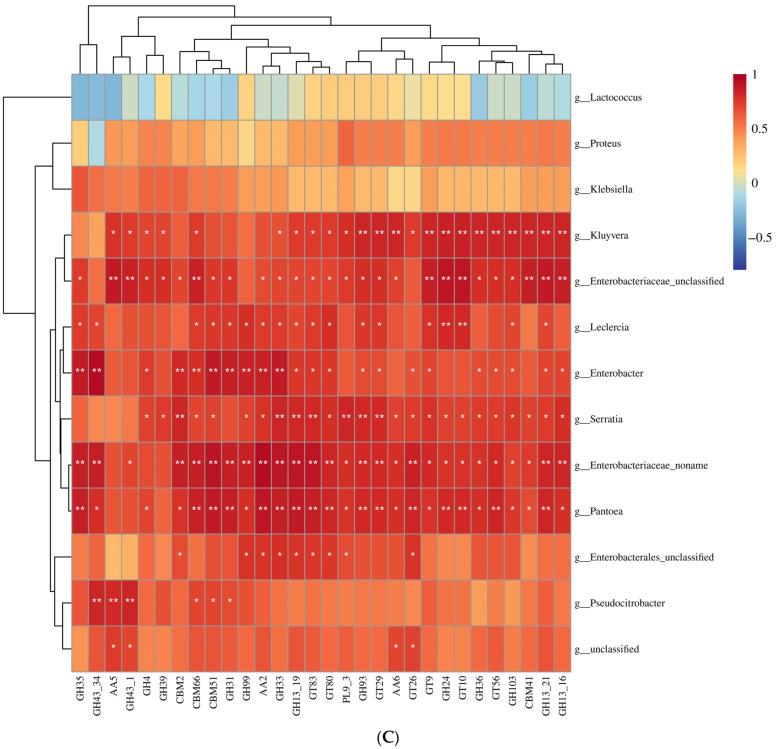
Comparison of the genes assigned to the CAZymes database. (**A**) Classification of CAZyme families in the samples. (**B**) The different abundant genes assigned to CAZy level 2. (**C**) Correlation analysis between microbiota and CAZymes (*, *p* < 0.05; **, *p* < 0.01).

Based on the analysis of carbohydrate metabolism in Ensifera using the KEGG database, we performed a detailed pathway analysis using the carbohydrate-active enzymes from the CAZymes database. A correlation between the gut microbiota and CAZymes was established. The annotated genes belonged to six classes of CAZymes, mainly glycosyl transferases (GTs), glycoside hydrolases (GHs), and carbohydrate esterases (CEs). GTs involved in catalyzing the transfer of sugar moieties forming glycosidic bonds and GHs involved in the hydrolysis of glycosidic bonds and carbohydrate esters were the most abundant CAZymes, which combined contributed 83.67%, 83.65%, and 94.78% of the abundance of Mec, Oce, and Gry, respectively (Figure 5A). Interestingly, CEs represented a small amount in Mec and were almost absent in Gry, whereas their abundance was 11.77% in Oce.

A deeper analysis of the abundance and diversity of CAZymes (CAZy level 2) revealed that GT1, GH17, GH38, and CE8 were the dominant enzyme families in Oce through pairwise comparison (Welch’s *t*-test, *p* < 0.01), while CE3 and CBM10 were the dominant enzyme families in Mec (Welch’s *t*-test, *p* < 0.05) (Figure 5B). Notably, the CAZymes family with significant differences in Mec was a unique enzyme family. No enzyme family significantly higher than the other species was found in Gry (Table 4). The enzyme families with the highest abundance among the samples were GT1, GT2, GT4, GT47, GH3, and CE8. GT2, as an enzyme with high abundance, mainly existed in Mec and Gry. It was found in all types of microorganisms, but mainly existed in bacteria. The representative enzyme was cellulose synthase (EC 2.4.1.12), which was involved in cellulose synthesis, and chitin synthase (EC 2.4.1.16), which converted UDP-N-acetyl-α-D-glucosamine into chitin and UDP. CE8, the enzyme family with the second highest abundance in Oce, was almost absent in Gry, as it mainly existed in bacteria and eukaryotes. The representative enzyme was pectin methyl esterase (EC 3.1.1.11), which catalyzed pectin hydrolysis to produce pectinic acid and methanol. Meanwhile, GH3, with β-glucosidase as the representative enzyme, was involved in the hydrolysis β-D-glucosyl residues with the release of β-D-glucose.

In order to identify what microbial species play an essential role in the carbohydrate active enzyme families, we screened 109 genes with an identity of 100% in the CAZymes database and analyzed the correlation with the CAZy level 2 (Figure 5C). The results show that *Kluyvera*, Enterobacteriaceae_unclassified, *Enterobacter*, Enterobacteriaceae_noname, and Pantoea were positively correlated microorganisms, and *Lactococcus* was a negatively correlated microorganism. These five microorganisms belonged to a bacterial genus of Mec with high abundance, but their functions associated with the CAZymes database were not highly abundant or unique.

## 4. Discussion

Feeding characteristics were the primary factor affecting the structure of the gut bacterial communities of Ensifera [36], and they significantly affected the gut microbial composition of other organisms [55,56,57,58]. This study explored the microbial community composition and function in three representatives of Ensifera mediated by feeding characteristics to deeply analyze the interaction between feeding habits and the gut microbiota. 

Previous studies found that the taxa with complex feeding habits had a higher gut bacterial diversity than those with single feeding habits [25,27]. This was also the result of our previous study on the gut bacterial diversity of Ensifera [36]. However, in this comprehensive study on gut bacteria, eukaryotes, viruses, and archaea by shotgun metagenomic sequencing, it was found that, although the omnivorous Gry had the most significant number of reads and contigs, its microbial diversity was not the highest. On the contrary, the herbivorous Mec had the highest gut microbial diversity. LEfSe analysis found that bacteria were the main biomarkers of the omnivorous Gry, and archaea and eukaryotes played an important role in Mec. Therefore, we speculate that eukaryotes, archaea, and viruses may have high diversity in the herbivorous Mec. Although the gut microbiota of the herbivorous Mec was highly diversified, we did not find a biomarker in the bacterial communities with the highest abundance.

Proteobacteria was involved in degrading cellulose substances in the host gut [59]. Similarly, we also found such characteristics in Ensifera, which showed that Proteobacteria in the herbivorous Mec accounted for about 80% of the total microbiota abundance. Among these species, bacteria were the dominant microbiota, followed by viruses, eukaryotes, and archaea. Notably, the abundance of viruses was higher than that of eukaryotes in the herbivorous Mec and omnivorous Gry. Moreover, it was previously reported that honeybee gut viruses were a microbial population second only to bacteria [60]. Conversely, viruses in the carnivorous Oce were almost absent, and possibly implied a high abundance of gut viruses in plant-feeding Ensifera. In the previous study, Cyanobacteria mainly existed in herbivores compared with other feeding habits [61]; and could even be used as a biomarker for herbivorous insects. Interestingly, with the more accurate shotgun metagenomic sequencing [5,62], the abundance of Cyanobacteria decreased significantly and was almost undetected in the carnivorous Oce and omnivorous Gry.

Diet was the main driving factor for the functional composition of the gut microbial community [27,45]. To explore the functional characteristics of the gut microbiota in Ensifera, we annotated the metagenomic genes using the KEGG and CAZymes databases [61,63,64]. Interestingly, although the herbivorous Mec had a high abundance of genes in the carbohydrate metabolism pathway, we also found that the carnivorous Oce had the most substantial ability to convert maltose into glucose and that the omnivorous Gry had the most substantial ability to convert cellulose into glucose, in the carbohydrate metabolism pathway. This indicated that Ensifera, with different feeding habits, had unique methods of converting polysaccharides into monosaccharides to obtain energy. However, the herbivorous Mec had a more vital ability to metabolize carbohydrates. Moreover, the carnivorous Oce did not show an advantage in the lipid metabolism pathway, which was far from our thoughts. Importantly, we did not find a high abundance of genes related to food digestion and absorption in the omnivorous Gry. It was speculated that the specificity of genes involved in nutrient metabolism in Ensifera with a single feeding habit was higher than that with a broad-spectrum feeding habit. Ensifera, with a single feeding habit, had a more vital ability to obtain nutrition from food. However, we found a high abundance of resistance genes in Gry, suggesting that the omnivorous Gry’s defense mechanism was better than that of herbivorous and carnivorous Ensifera species.

In this study, GTs were the most abundant enzymes in all samples, which was inconsistent with some animal reports that GHs were the most highly expressed enzymes [49,65]. GTs played a role in the biosynthesis of disaccharides, oligosaccharides, and polysaccharides, catalyzed the transfer of sugar groups to aglycones, and were very important for synthesizing many natural products [66]. GT2, with the high abundance of chitin synthase, was mainly found in the herbivorous Mec, as chitin mainly existed in the epidermis of insects, which indicated that the herbivorous Mec could digest not only cellulose but also chitin in the insect epidermis. CE8, with the high abundance of pectin methyl esterase, was mainly found in the carnivorous Oce, as pectin primarily existed in the plant cell wall and inner layer [67], which was consistent with the conclusion in the KEGG database that the carnivorous Oce can convert maltose into glucose to obtain nutrition and energy by digesting food derived from plants. Meanwhile, GH3 with the representative enzyme β-glucosidase involved in the hydrolysis release of β-D-glucose, consisting of the star and sucrose metabolic pathways of the KEGG database, was found in Gry, which could efficiently convert glucose. The above results show that in the gut of Ensifera species with a specific feeding habit, the genes of metabolic pathways and enzyme families related to the feeding habit have a high abundance, and those related to energy and nutrient digestion and absorption may also have a high abundance. It was speculated that Ensifera might have to retain or evolve functions in order to adapt to extreme environments [58]. 

We found that the abundance of genes related to the metabolic pathway of the omnivorous Gry in drug resistance was significantly higher than that of the other species. Gry usually lives in soil; however, we still do not know the role of such a high-abundance metabolic pathway in its life activities. Although we detected the most significant number of contigs and many unique microbial populations at the phylum and species levels, we did not find a good performance in Gry’s unique functions of gut microbiota. For example, a low abundance of KEGG metabolic pathways related to food digestion and absorption was found, and no unique CAZyme family was found. This might indicate that the more complex the feeding behavior of Ensifera, the worse the functional specificity related to the feeding behavior of its gut microbiota. Furthermore, microbiota with a high correlation with the CAZymes database had a high abundance, but the enzyme families associated with the microbiota were not highly abundant or unique. This might mean that the functional microbiota in the gut was not necessarily high-abundance microbiota [65,68], but maybe some low-abundance or unique microbiota.

## 5. Conclusions

Our results show significant differences in the gut microbial community of Ensifera are mediated by feeding behavior and that the main functions of the gut microbiota were consistent with feeding characteristics. Specifically, the microbial community diversity of herbivorous Ensifera species was higher than that of the omnivorous and carnivorous species. At the same time, it was found that the abundance and specificity of the microbial population related to feeding habits in omnivorous Ensifera species was low, indicating that Ensifera species with a single feeding habit had a more vital ability to obtain nutrition from food. We also found that the gut microbiota associated with a higher abundance of metabolic pathways and carbohydrate active enzyme families were highly correlated with feeding characteristics. However, some microorganisms that had nothing to do with feeding characteristics, but were related to energy acquisition and nutrient absorption, also had a high abundance. In addition, gut microbiota with a low abundance may play an essential role in the life activities of Ensifera.

## Figures and Tables

**Table 1:** Overview of sample information.

Taxonomy		Feeding Habits	Location		Abbreviations
Family	Species		County/Mountain, Province	Geographic Coordinates	
Tettigoniidae	*Mecopoda niponensis*	Herbivore	Tianmu Mountains National Nature Reserve, Zhejiang	30°35′ N 119°43′ E	Mec
Gryllacrididae	*Ocellarnaca emeiensis*	Carnivore	Daming Mountains National Nature Reserve, Guangxi	23°52′ N 108°34′ E	Oce
Gryllotalpidae	*Gryllotalpa orientalis*	Omnivore	Quyang County, Hebei	38°59′ N 114°78′ E	Gry

**Table 2:** Top 4 abundances in the gut microbiota at the phylum and species levels of samples with different feeding habits.

Samples		*Gryllotalpa orientalis*		*Mecopoda niponensis*		*Ocellarnaca emeiensis*	
Microbial Taxonomy		Phylum/%	Species/%	Phylum/%	Species/%	Phylum/%	Species/%
**Identification** **of microbiota**	Top1	Firmicutes/47.09	*Intestinimonas massiliensis*/42.12	Proteobacteria/77.65	*Leclercia adecarboxy lata*/12.80	Proteobacteria/40.34	*Lactococcus* *lactis*/23.82
	Top2	Proteobacteria/31.80	*Acinetobacter* *baumannii*/15.42	Mucoromycota/7.43	*Kluyvera cryocrescens*/9.51	Firmicutes/20.04	*Acinetobacter baumannii*/20.86
	Top3	Bacteroidetes/8.69	*Cotesia sesamiae bracovirus*/10.82	Basidiomycota/2.94	*Pseudomonas aeruginosa*/9.16	Basidiomycota/14.64	*Rhizophagus irregularis*/8.16
	Top4	Candidatus_Tectomicrobia/2.61	*Pseudomonas* *aeruginosa*/7.8	Firmicutes/2.51	*Acinetobacter baumannii*/8.47	Mucoromycota/7.01	*Solemya velum gill symbiont*/8.14

**Table 3:** The percent of gut microbiota was assigned to biological functions.

KEGG Pathway	Percent of Genes (%)		
	Mec	Oce	Gry
Organismal systems	0.00	0.06	0.00
Metabolism	72.78	65.35	10.95
Human diseases	1.86	2.03	83.60
Genetic information processing	8.66	17.58	4.00
Environmental information processing	11.22	10.49	0.79
Cellular processes	5.48	4.49	0.66

**Table 4:** Comparison of CAZyme families in the three species.

Groups	High Abundance CAZymes		Significantly Different CAZymes		Unique CAZymes	
	CAZyme Family	Activities in Family	CAZyme Family	Activities in family	CAZyme Family	Activities in Family
Mec	GT47	heparan β-glucuronyltransferase (EC 2.4.1.225); xyloglucan β-galactosyltransferase (EC 2.4.1.-)	CE3	acetyl xylan esterase (EC 3.1.1.72)	CE3	acetyl xylan esterase (EC 3.1.1.72)
	GT2	cellulose synthase (EC 2.4.1.12); chitin synthase (EC 2.4.1.16)	CBM10	cellulose-binding function	CBM10,	cellulose-binding function
	GT4	sucrose synthase (EC 2.4.1.13); sucrose-phosphate synthase (EC 2.4.1.14)			GH5_18	b-mannosidase (EC 3.2.1.25)
					GH5_13	b-D-galactofuranosidase (EC 3.2.1.146);a-L-arabinofuranosidase (EC 3.2.1.55)
					GH100	alkaline and neutral invertase (EC 3.2.1.26)
Oce	GT47	heparan β-glucuronyltransferase (EC 2.4.1.225); xyloglucan β-galactosyltransferase (EC 2.4.1.-)	CE8	pectin methylesterase (EC 3.1.1.11)		
	CE8	pectin methylesterase (EC 3.1.1.11)	GT1	UDP-glucuronosyltransferase (EC 2.4.1.17); zeatin O-β-xylosyltransferase (EC 2.4.2.40)		
	GT1	UDP-glucuronosyltransferase (EC 2.4.1.17); zeatin O-β-xylosyltransferase (EC 2.4.2.40)	GH38	α-mannosidase (EC 3.2.1.24); mannosyl-oligosaccharide α-1,2-mannosidase (EC 3.2.1.113)		
			GH17	glucan endo-1,3-β-glucosidase (EC 3.2.1.39); licheninase (EC 3.2.1.73)		
Gry	GT2	cellulose synthase (EC 2.4.1.12); chitin synthase (EC 2.4.1.16)				
	GH3	β-glucosidase (EC 3.2.1.21); xylan 1,4-β-xylosidase (EC 3.2.1.37)				
	GT47	heparan β-glucuronyltransferase (EC 2.4.1.225); xyloglucan β-galactosyltransferase (EC 2.4.1.-)				

## Data Availability

The datasets presented in this study can be found in NCBI under project number PRJNA762197.

## Supplementary Materials

The following supporting information can be downloaded at: https://www.mdpi.com/article/10.3390/insects13080719/s1. Figure S1: A stacked bar chart revealed the relative abundance of gut viruses and archaea composition at (A,C) phylum and (B,D) species levels. The results show the phylum and species of the gut microbiota with the highest abundance; Figure S2: Distribution diagram of the LEfSe analysis based on the LDA score of (A) eukaryote and (B) viruses to screen the biomarkers; Figure S3: Pathway annotation comparisons of the genes assigned to the KEGG database. (A) Gene abundance comparison in galactose metabolism pathway. (B) Gene abundance comparison in arginine biosynthesis pathway. (C) Gene abundance comparison in phenylalanine, tyrosine and tryptophan biosynthesis pathway. (D) Gene abundance comparison in beta lactam resistance pathway. Note: the genes belonging to the three samples with significant differences in the metabolic pathway map are marked by color, in which Mec, Oce, and Gry are from left to right. The color from red to green represents the gene abundance from high to low; Table S1: raw and clean reads from the analyzed samples; Table S2: general assembly and mapping statistics for samples; Table S3: relative abundance of classified gut microbiota in samples.
