# Supplementary material for: The Role of Feeding Characteristics in Shaping Gut Microbiota Composition and Function of Ensifera (Orthoptera)
Source: Insects. 2022 Aug 10;13(8):719. doi: 10.3390/insects13080719 (PMC9409189; doi:10.3390/insects13080719)
Supplement: Supplementary file 1 [file insects-13-00719-s001.zip › Table S1.pdf]

Table S1. Raw and clean reads from the analyzed samples.

Sample	Reads		Clean/%	Q30/%	GC/%	Host genome rate/%
	Raw Data	Clean Data				
Mec	79,442,693	75,163,887	94.66	93.76	41.08	1.72
Oce	82,575,455	80,072,494	96.96	94.37	38.33	1.16
Gry	85,662,373	81,561,753	95.24	93.51	41.89	3.29
