# Supplementary material for: The Role of Feeding Characteristics in Shaping Gut Microbiota Composition and Function of Ensifera (Orthoptera)
Source: Insects. 2022 Aug 10;13(8):719. doi: 10.3390/insects13080719 (PMC9409189; doi:10.3390/insects13080719)
Supplement: Supplementary file 1 [file insects-13-00719-s001.zip › Table S2.pdf]

Table S2. General assembly and mapping statistics for samples by shotgun.

Sample	No.of contigs	Largest contig (bp)	Total length (bp)	N50	N75	L50	L75	GC%
Mec	71,476	119,727	53,110,320	673	559	24,300	46,242	43.64
Oce	129,921	197,741	90,019,027	627	548	48,056	86,771	39.31
Gry	417,677	126,418	343,576,907	730	584	130,574	259,485	41.93
