# Supplementary material for: The Role of Feeding Characteristics in Shaping Gut Microbiota Composition and Function of Ensifera (Orthoptera)
Source: Insects. 2022 Aug 10;13(8):719. doi: 10.3390/insects13080719 (PMC9409189; doi:10.3390/insects13080719)
Supplement: Supplementary file 1 [file insects-13-00719-s001.zip › Table S3.pdf]

Table S3. Relative abundance of classified gut microbiota in samples.

Domain	Abundance of gut microbiota		
	**Mec（%）**	**Oce（%）**	**Gry（%）**
Bacteria	81.91	88.71	86.03
Eukaryote	6.52	9.93	5.61
Viruses	11.19	1.25	8.22
Archaea	0.37	0.10	0.13
