# Supplementary figures and images for: The Role of Feeding Characteristics in Shaping Gut Microbiota Composition and Function of Ensifera (Orthoptera)
Source: Insects. 2022 Aug 10;13(8):719. doi: 10.3390/insects13080719 (PMC9409189; doi:10.3390/insects13080719)

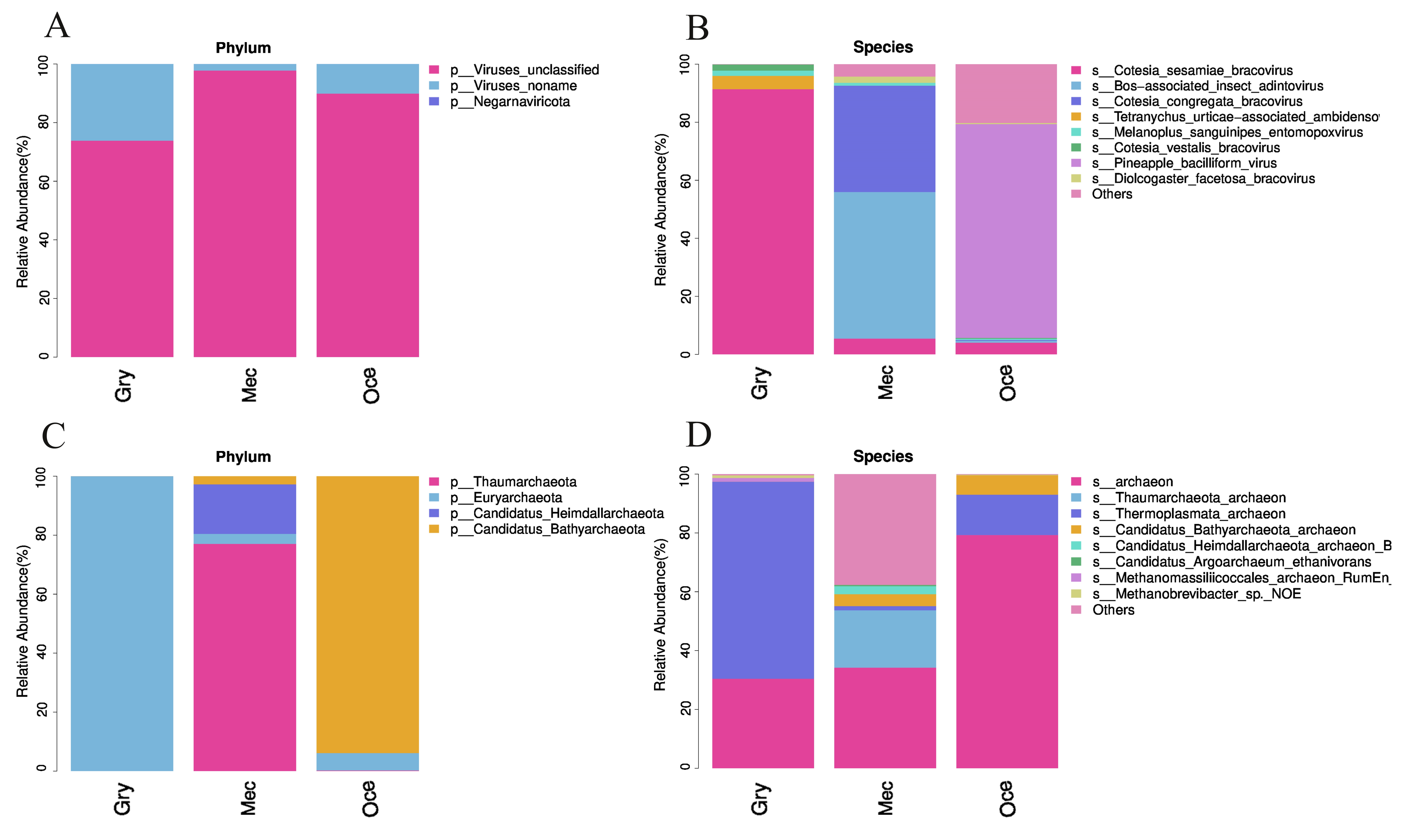

Supplement: Supplementary file 1 [file insects-13-00719-s001.zip › Figure S1.jpg]

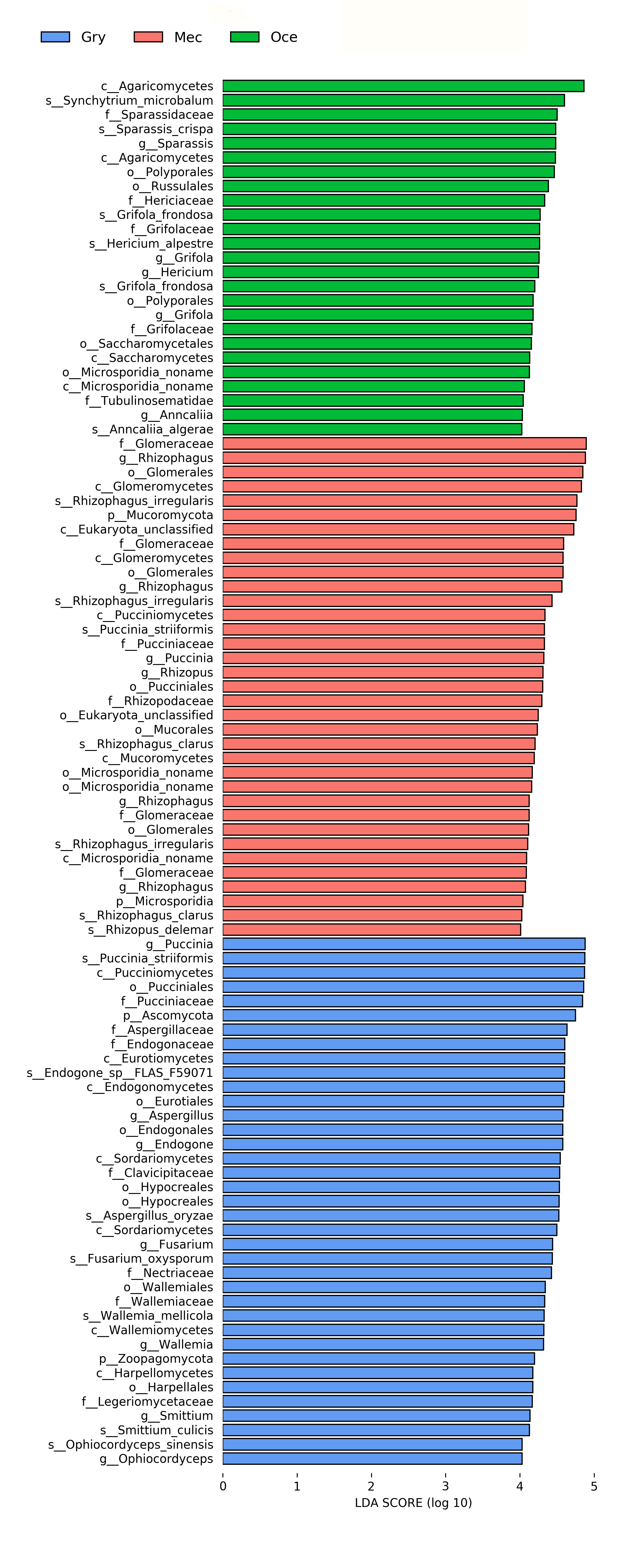

Supplement: Supplementary file 1 [file insects-13-00719-s001.zip › Figure S2A.jpg]

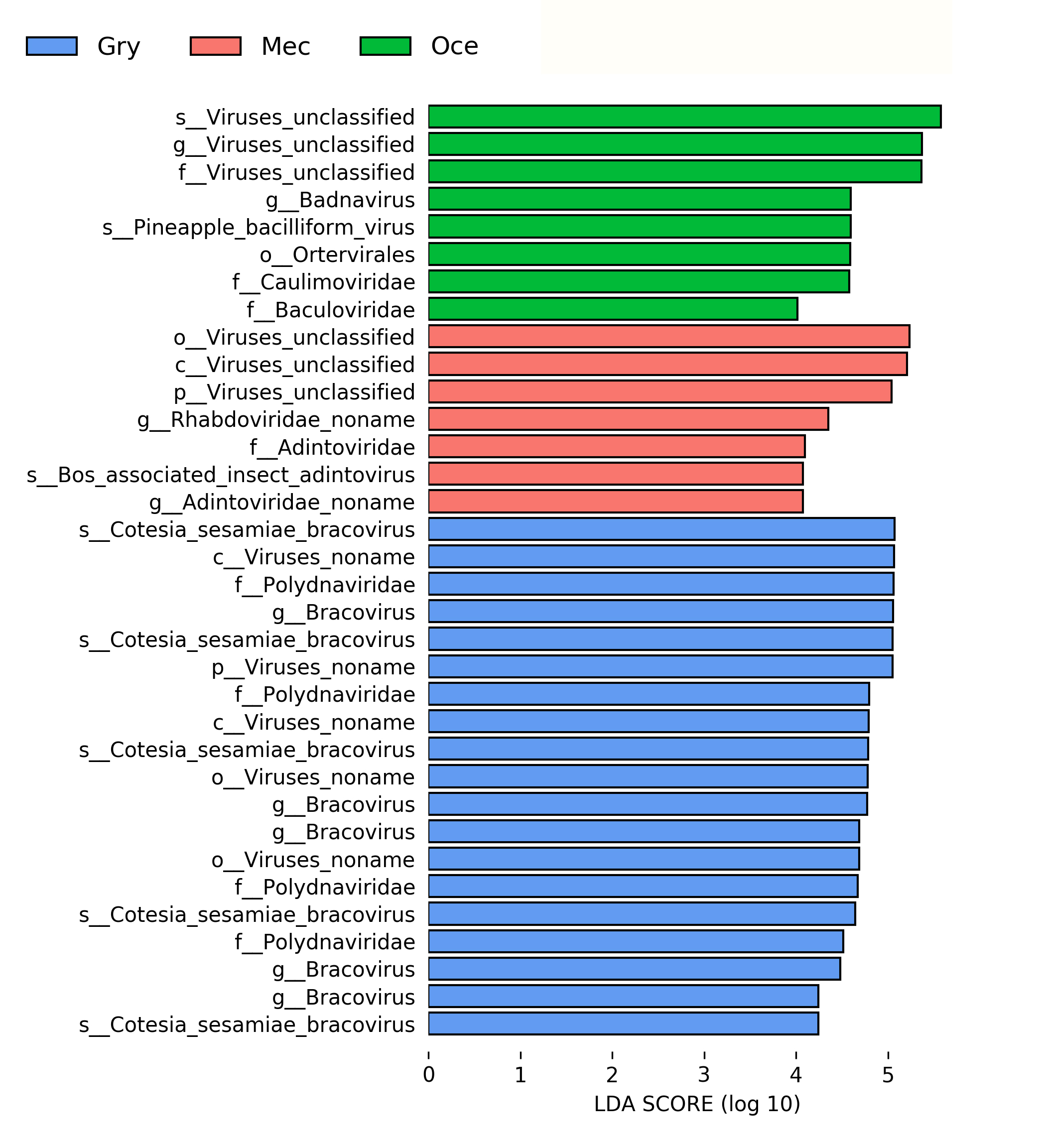

Supplement: Supplementary file 1 [file insects-13-00719-s001.zip › Figure S2B.jpg]

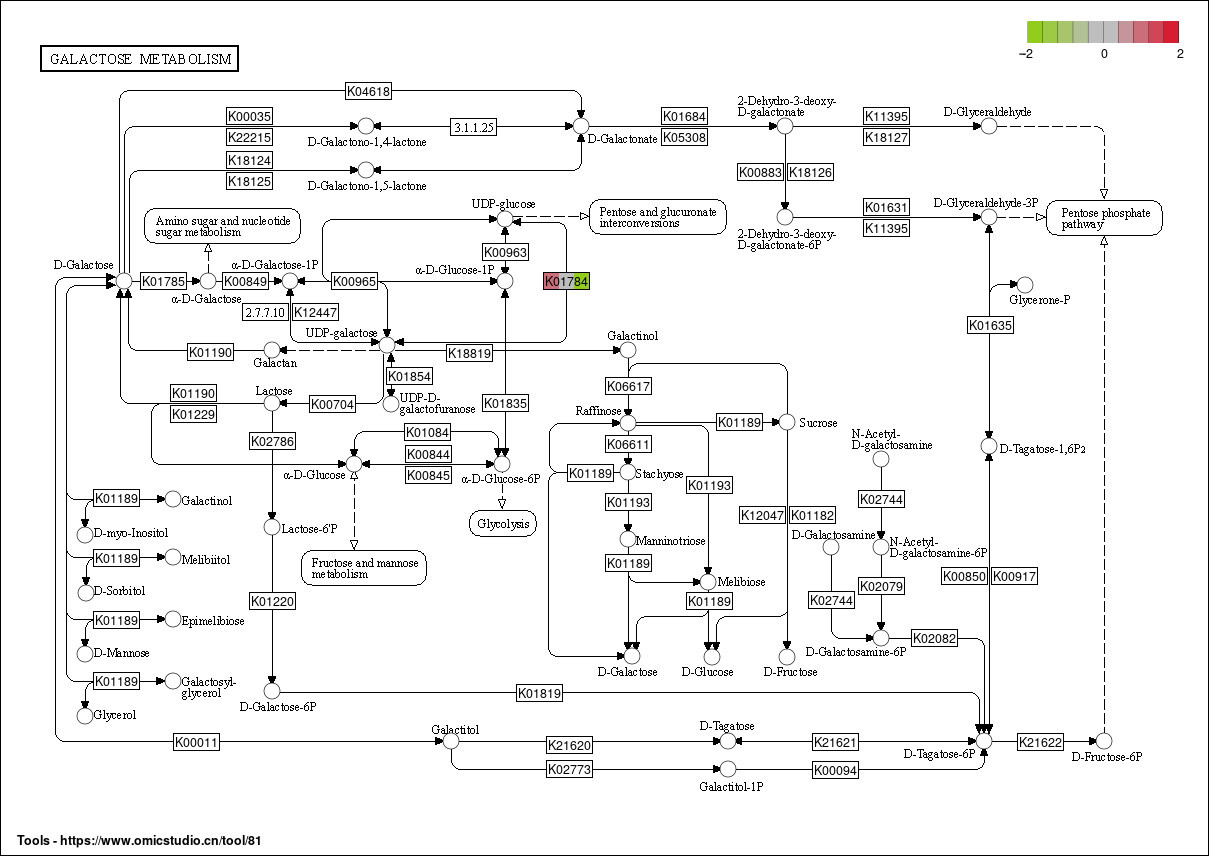

Supplement: Supplementary file 1 [file insects-13-00719-s001.zip › Figure S3A.jpg]

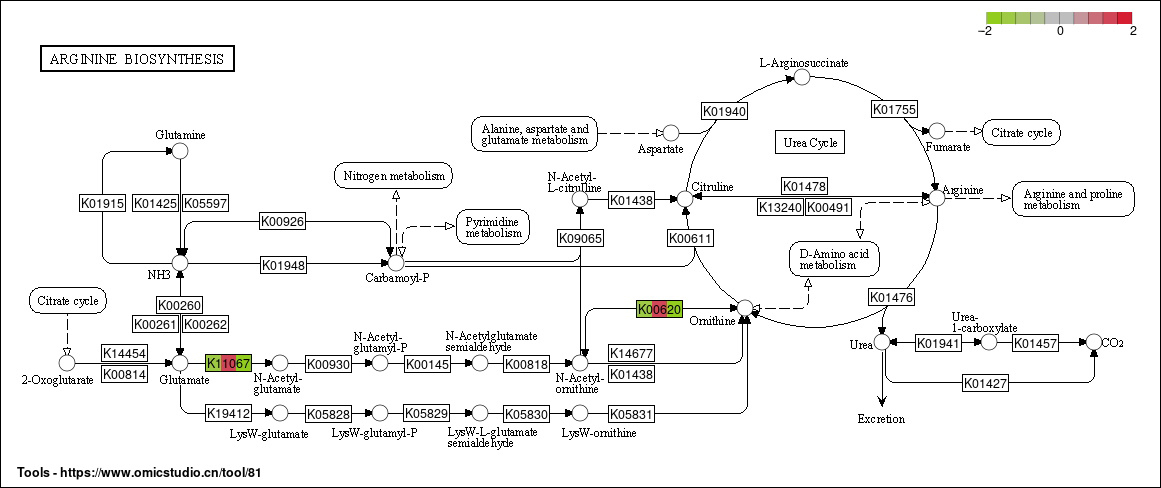

Supplement: Supplementary file 1 [file insects-13-00719-s001.zip › Figure S3B.jpg]

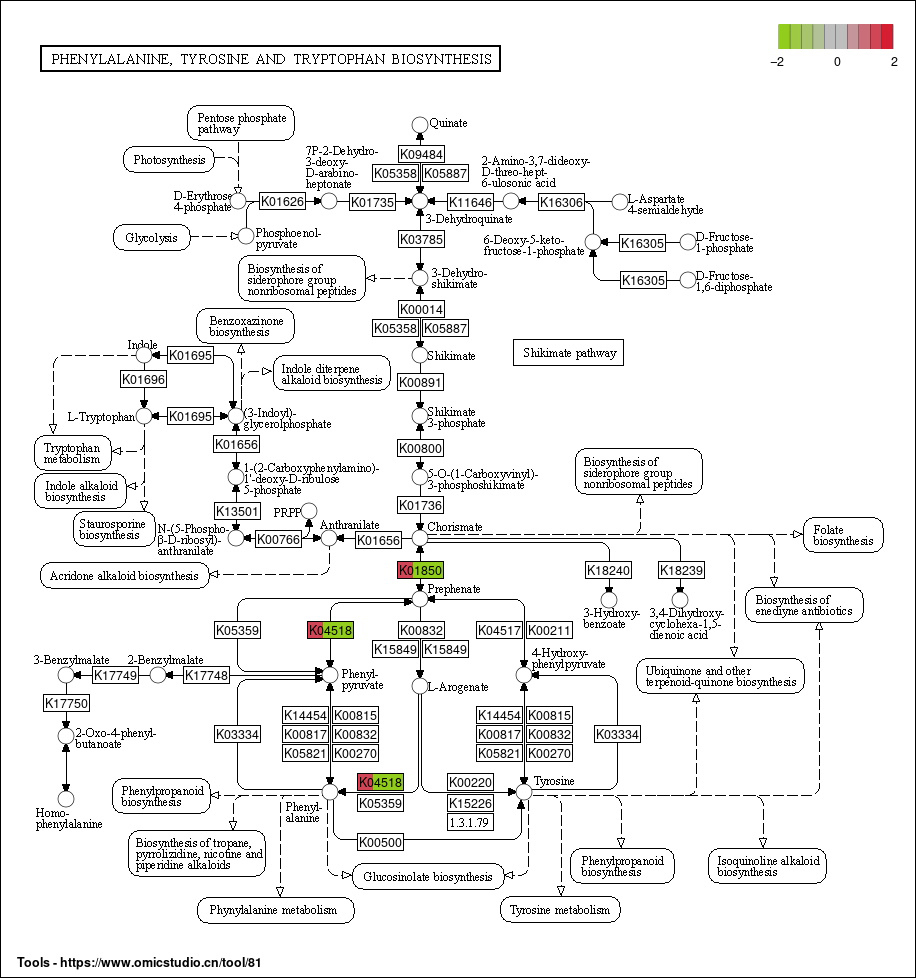

Supplement: Supplementary file 1 [file insects-13-00719-s001.zip › Figure S3C.jpg]

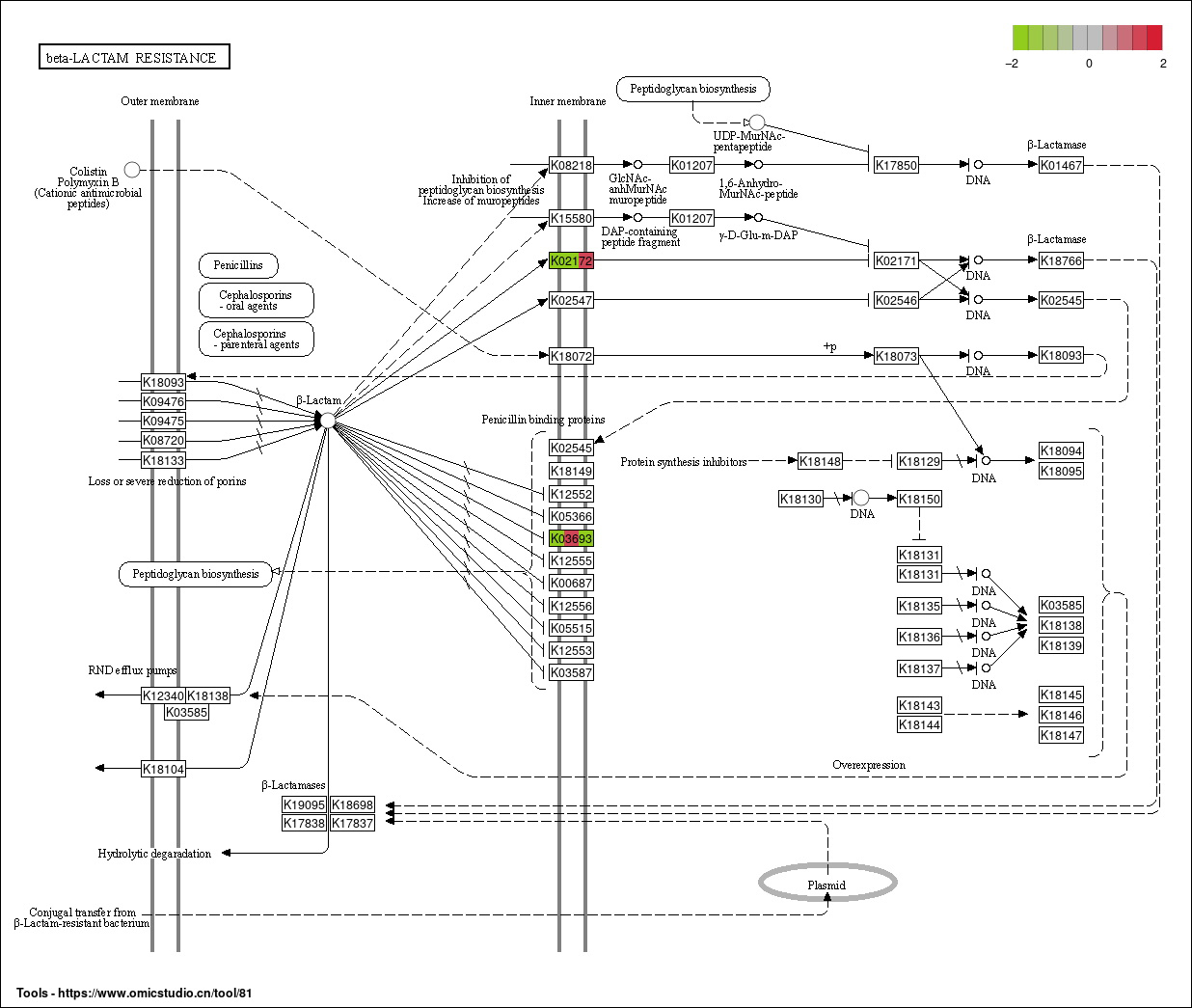

Supplement: Supplementary file 1 [file insects-13-00719-s001.zip › Figure S3D.jpg]
